# Cryo-EM structure of the homohexameric T3SS ATPase-central stalk complex reveals rotary ATPase-like asymmetry

**DOI:** 10.1038/s41467-019-08477-7

**Published:** 2019-02-07

**Authors:** Dorothy D. Majewski, Liam J. Worrall, Chuan Hong, Claire E. Atkinson, Marija Vuckovic, Nobuhiko Watanabe, Zhiheng Yu, Natalie C. J. Strynadka

**Affiliations:** 1 Department of Biochemistry and Molecular Biology and the Center for Blood Research, University of British Columbia, Vancouver, BC Canada; 2 HRMEM Facility, University of British Columbia, Vancouver, BC Canada; 3 CryoEM Shared Resources, Howard Hughes Medical Institute, Janelia Research Campus, Ashburn, VA USA; 4 Present Address: Merck & Co., Department of Biochemical Engineering and Structure, 2000 Galloping Hill Road, Kenilworth, NJ 07033 USA

## Abstract

Many Gram-negative bacteria, including causative agents of dysentery, plague, and typhoid fever, rely on a type III secretion system – a multi-membrane spanning syringe-like apparatus – for their pathogenicity. The cytosolic ATPase complex of this injectisome is proposed to play an important role in energizing secretion events and substrate recognition. We present the 3.3 Å resolution cryo-EM structure of the enteropathogenic *Escherichia coli* ATPase EscN in complex with its central stalk EscO. The structure shows an asymmetric pore with different functional states captured in its six catalytic sites, details directly supporting a rotary catalytic mechanism analogous to that of the heterohexameric F_1_/V_1_-ATPases despite its homohexameric nature. Situated at the C-terminal opening of the EscN pore is one molecule of EscO, with primary interaction mediated through an electrostatic interface. The EscN-EscO structure provides significant atomic insights into how the ATPase contributes to type III secretion, including torque generation and binding of chaperone/substrate complexes.

## Abstract (web summary)

Many Gram-negative bacteria rely on a type III secretion system (T3SS) for their pathogenicity. Here authors present the cryo-EM structure of the E.coli T3SS ATPase-central stalk complex, which forms a homohexameric, asymmetric pore with different functional states.

## Introduction

Rotary ATPases are a biologically important and well-conserved protein family, fuelling vital life processes from archaea to humans. One of the earliest examples of molecular machines, their well-studied mechanism of ATP binding and hydrolysis fuels conformational changes to generate torque. The related F- and V-ATPases are composed of a soluble catalytic complex that can synthesize or hydrolyse ATP (F_1_/V_1_), which is coupled to a transmembrane proton (or sodium) channel (F_o_/V_o_). These motors have opposite roles depending on their cellular context: F-ATPases use membrane potential to rotate the F_o_ complex and synthesize ATP at the coupled F_1_ domain, while V-ATPases use energy derived from ATP hydrolysis to pump protons across the membrane and acidify intracellular compartments such as vacuoles.

The broad F_1_/V_1_-ATPase family—with characteristic Rossmann fold, Walker A and B motifs and hexameric stoichiometry—also encompasses distant relatives including ATPases associated with the bacterial injectisome and flagellum^1^. These two proteinaceous assemblies, involved in virulence and motility, use an evolutionarily related type III secretion system (T3SS; referred to here as *f*T3SS (flagellar) and *v*T3SS (virulence injectisome)) to secrete self-assembly and host-cell manipulating substrates. The T3SS ATPase homologues are made distinct by their homohexameric construction, a presumed ancestral precursor to the heterohexameric rotary F_1_/V_1_-ATPases. Homology of the type III secretion (T3S) ATPases to the catalytic F_1_/V_1_ subunits was first predicted based on sequence comparison^2,3^, and subsequently demonstrated with monomeric structures of the *f*T3SS ATPase FliI (PDB: 2DPY)^4^ and the enteropathogenic (EPEC) *Escherichia coli* *v*T3SS ATPase EscN (2OBL, 2OBM)^5^. Further evolutionary similarities were found in soluble accessory components of the F_1_/V_1_-ATPases, with sequence and structural homology demonstrated for the peripheral^6,7^ and central^8–12^ stalks. Accessory subunits form the foundation of ATPase function: the central stalk acts as a rotor and couples the F_1_/V_1_ and F_o_/V_o_ complexes, while the peripheral stalks act as stators to prevent rotation of the catalytic subunits. The evolutionary relationship and conserved elements of this core complex raise the intriguing possibility that T3S may utilize a related torque-generating motor in bacterial secretion.

The injectisome is a highly complex nanomachine that secretes specific bacterial effector proteins directly into the cytoplasm of infected host cells, a process that allows subsequent subversion of cell signalling to the pathogen’s advantage. The injectisome is essential to the virulence of a broad range of pathogens, including EPEC/enterohaemorragic, *Salmonella*, *Shigella*, *Pseudomonas*, *Yersinia*, *Chlamydia* and *Bordetella*: causative agents of food poisoning, dysentery, nosocomial infections, plague, sexually transmitted infections and whooping cough. Although the T3S-delivered effectors vary amongst the different pathogens, the secretion apparatus itself is well conserved and thus of interest as a multivalent target of anti-virulence therapeutics and vaccines.

The injectisome must overcome the significant hurdles of passaging effectors through the inner and outer membranes of the Gram-negative envelope, the intervening cell-wall peptidoglycan mesh, the extracellular space (~90 nm in EPEC) and the host-cell membrane. The major structural scaffold of the injectisome is the basal body, comprising concentric, highly oligomerized membrane-spanning protein rings that pass through the Gram-negative envelope. The attached extracellular needle terminates with a translocon pore-forming complex that punctures the host-cell membrane, creating a continuous channel from pathogen to host. This assembly results in the characteristic syringe-like appearance, with a wide cylindrical body adjoining a hollow needle of ~20 Å inner diameter through which effector proteins can pass in a semi-folded state (for a T3SS review, see Deng et al.^13^). Substrate selection occurs in the cytoplasm and inner membrane, mediated by the export gate subcomplex (EscRSTUV in EPEC, and FliPQR, FlhB, FlhA in flagella) and the cytoplasmic ATPase complex (ATPase EscN, peripheral stalk EscL, and central stalk EscO; flagellar FliI, FliH and FliJ, respectively). Together, these components must presumably work to select, prepare and secrete effector proteins though the injectisome.

The T3SS has co-opted an early ancestor of F_1_/V_1_-ATPases for its distinct, customized action. The energy for secretion was historically proposed to solely derive from the T3S ATPase^14^, but was subsequently shown in *f*T3S to depend on proton motif force (PMF)^15,16^, mediated in part by the partnering export gate component FlhA (homologue of EPEC EscV)^17,18^. The ATPase has been proposed to specifically enhance PMF-induced secretion efficiency^18^, as well as to play a role in effector targeting, release from chaperones and unfolding prior to secretion^19–22^. Most T3S effectors rely on protective chaperones to deliver them in a partially unfolded state to the cytosolic complex^23^ and it has been demonstrated that the *v*T3SS ATPase can dissociate T3S effector cargo from its cognate chaperone^20–22^. It is hypothesized that the partially unfolded effector (including structural elements as large as single helices^24^) is subsequently passaged into the adjacent export gate protein pore (EscV in EPEC) and funnelled into the hollow T3SS needle for secretion.

Although predicted to function as a hexamer by analogy to the rotary ATPases, all structures to date of T3SS ATPase orthologues^4,5,7,25–27^ have been monomeric, resulting in a poor understanding of the precise atomic details of oligomerization and cooperative catalytic function in these homohexameric variants. Similarly, little is known about the ATPase-central stalk interaction and how that relates to its role in T3S. To address these questions, we determine here the cryo-electron microscopy (cryo-EM) structure of a homohexameric T3S ATPase EscN in complex with its central stalk protein EscO. The 3.3 Å resolution is sufficient to trace conformational differences, model amino acid side chains and observe bound ligands and metal ions. Remarkably, EscN’s asymmetric hexamer reveals similarity to the rotary ATPases that extends beyond the monomer, despite not sharing their heterohexameric construction. The different catalytic states observed in its active sites and relative disposition of the central stalk are in close agreement with previous structures of various assembled F_1_ and V_1_ heterohexameric ATPases, showing conservation of the necessary conformational elements required for a rotary ATPase mechanism. This resulting structural evidence for EscN/EscO torque generation brings us closer to unravelling the mechanism of ATPase action in T3S substrate selection and energetics.

## Results

### Cryo-EM analysis of the EscN-EscO complex

Various constructs of EPEC EscN were screened for expression and purification, with the aim of isolating a stable oligomer for structural characterization. The most stable construct, EscN^29–446^, was predominantly monomeric but readily formed oligomers in the presence of the transition state analogue Mg^2+^ADP-AlF_3_, according to size exclusion chromatography, glycerol gradient centrifugation and negative-stain EM (Supplementary Fig. 1). The catalytic activity of monomeric EscN was barely detectable at the 500 nM enzyme concentration used in our assay; however, pre-incubation with Mg^2+^ADP-AlF_3_ (but omission from the reaction) resulted in a substantial increase in enzymatic activity, supporting the formation of functional oligomers (Supplementary Fig. 2a, b). Full-length EPEC EscO was purified separately and found to further enhance EscN oligomerization and ATPase activity (Supplementary Fig. 2). Studies have shown the V-ATPase-central stalk protects A_3_B_3_ heterohexamers against ATP-induced dissociation^28^ and the *f*T3SS orthologue stabilizes the FliI ATPase complex^8^. Thus, the increased activity observed here may be due to similar stabilization of the EscN homohexamer. To isolate the complex, EscO was incubated in stoichiometric excess with EscN in the presence of Mg^2+^ADP-AlF_3_, with both co-sedimenting in a glycerol gradient. The complex formed visible rings (~10 nm diameter) in negative-stain EM and cryo-EM (Supplementary Figs 1c, 3a).

**Fig. 1:**
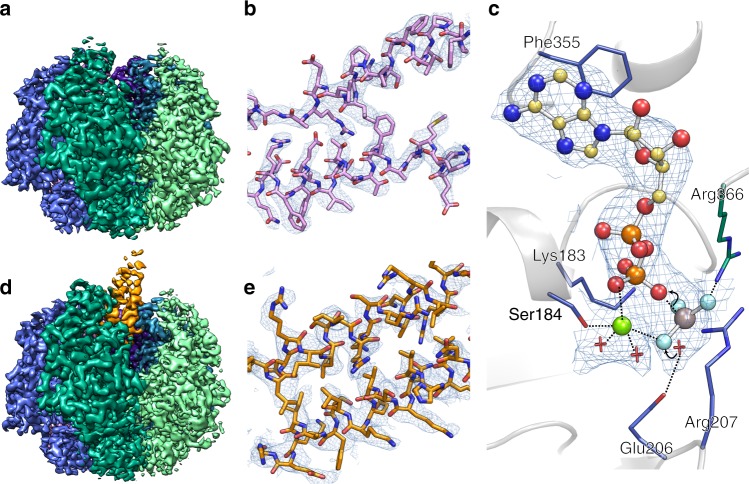
Cryo-EM density and resolution. Cryo-EM maps, coloured by subunit, of the **a** class one reconstruction comprising the EscN hexamer (without visible EscO density) at 3.34 Å resolution, and of the **d** class two reconstruction of the EscN-EscO complex at 3.29 Å resolution. Representative density is shown for **b** class one (EscN chain C residues 238–258 and 290–311) contoured at 6*σ* and **e** class two (EscO residues 1–19 and 103–122) contoured at 4*σ*. **c** Sample density of the class two catalytic site ligands ADP, AlF_3_ (grey and cyan), and Mg^2+^ (green), contoured at 4*σ*. Ligand coordination is represented with dotted lines, while the curved lines demarcate the reaction mechanism

**Fig. 2:**
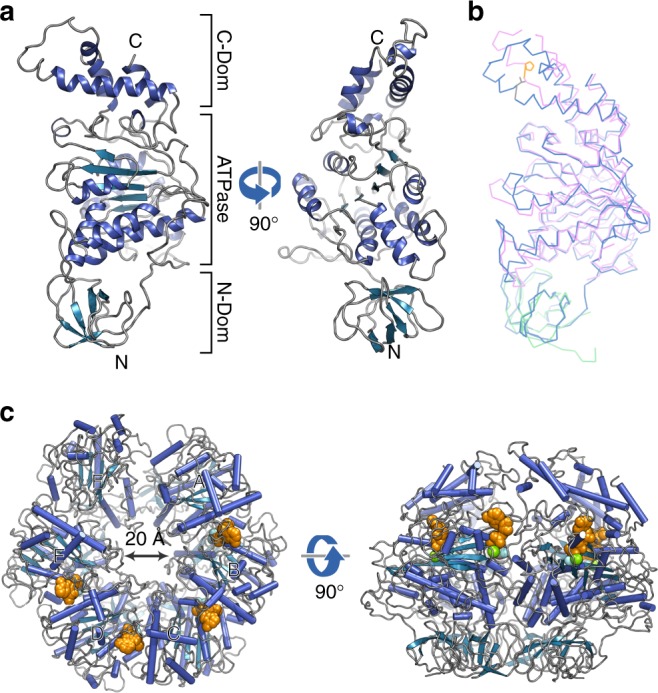
EscN structure. **a** Cartoon representation of the EscN chain D monomer from the class one cryo-EM model, shown from two views and coloured with α-helices in blue, β-sheets in teal and loops in grey. **b** Overlay of EscN chain D (blue) with EscN^103–446^ crystal structure (pink, PDB 2OBM) and homology model from FliI (PDB 5B0O) N-terminal domain (green), highlighting a highly similar structure save for differences in the C-terminal domain due to the Val393Pro mutation in 2OBM (shown in orange). **c** Overview of hexameric EscN class one model from top and side views, with modelled ADP (orange), Mg^2+^ (green) and aluminium fluoride (grey and cyan) shown as spheres

The EscN-EscO complex was subjected to single-particle cryo-EM, with 3D classification resulting in two primary classes that refined to high resolution (Supplementary Fig. 3). Class 1 (58,000 particles, 3.34 Å) reveals a homohexameric EscN pore lacking visible EscO density (Fig. 1a, b, Supplementary Fig. 3c–f), while class 2 (55,000 particles, 3.29 Å) looks similar but with additional α-helical density for approximately half of a single EscO molecule resolved (Fig. 1d–e, Supplementary Fig. 3h–l, Supplementary Fig. 4). Six copies of EscN were modelled into the class 1 reconstruction (EscN alone) guided by prior crystal structures of monomeric EscN and flagellar homologue FliI (PDBs 2OBM and 5B0O^5,7^, Fig. 2b). For clarity, we refer to the six chains of EscN as N_A_ through N_F_. The final refined model spans residues 35–446 for all chains, excepting unresolved loop 323–329 on chain N_F_ (Fig. 2a, c). The EscN model remained similar when refined into the class 2 map (Cα root-mean-square deviation (RMSD) of ~0.5 Å over 2130 residues), with unfilled density from two associated anti-parallel helices attributed to EscO. The quality of the side-chain density was sufficient for sequence assignment of EscO residues 0 (from affinity tag) to 30 and 91 to 122 out of a total of 125 residues (Fig. 1e, Supplementary Fig. 3i). The assigned EscO register fits well in the observed side-chain densities (EMRinger^29^ score for the EscO coordinates alone of 2.55), and agrees with prior experimental data^9^. In four of the six EscN inter-subunit active sites, the Mg^2+^ADP-AlF_3_ transition state analogue was clearly visible (Figs. 1c, 2c); the resolution was insufficient to differentiate between AlF_3_ and AlF_4_, so AlF_3_ was used as the more abundant species at pH 7.5^30^. The final overall models fit well to the EM data (with EMRinger scores of 3.5 and 3.4 for class 1 and 2) and good stereochemical indicators (Table 1).

**Table 1:** Cryo-EM data collection and refinement and validation statistics

	Class 1	Class 2
	(EMDB-9390)	(EMDB-9391)
	(PDB 6NJO)	(PDB 6NJP)
Data collection and processing		
Magnification	49,020	49,020
Voltage (kV)	300	300
Total dose (e^–^/Å^2^)	57.67	57.67
Dose per frame (e^–^/Å^2^)	1.92	1.92
Defocus range (μm)	−1.2 to −2.5	−1.2 to −2.5
Pixel size (super-resolution) (Å)	1.02	1.02
Symmetry imposed	C1	C1
Initial number of particles	376,000	376,000
Final number of particles	58,000	55,000
Map resolution (Å)	3.34	3.29
FSC threshold	0.143	0.143
Refinement		
Initial model used (PDB code)	2OBM, 5B0O	
Model resolution (Å)	3.34	3.29
FSC threshold	0.143	0.143
Map sharpening *B*-factor (Å^2^)	−98.5	−80.3
Model composition		
Non-hydrogen atoms	19,090	19,606
Protein residues	2466	2466
Ligands	12	12
* B*-factors (Å^2^)		
Protein	55.8	55.3
Ligand	24.3	26.8
R.m.s. deviations		
Bond lengths (Å)	0.01	0.0089
Bond angles (°)	1.45	1.4
Validation		
MolProbity score	1.78	1.77
Clashscore	5.91	6.23
Poor rotamers (%)	0.49	0.67
Ramachandran plot		
Favoured (%)	92.9	93.55
Allowed (%)	7.1	6.45
Disallowed (%)	0	0

### EscN forms an asymmetric ring conserved with F_1_/V_1_-ATPases

Each EscN monomer displays three sub-domains: the N-terminal oligomerization domain (residues 1–102), a cluster of curved β-sheets; the ATPase domain (103–372), made up of a central Rossmann fold with seven parallel β-strands flanked by four α-helices on one side and three on the other; and the C-terminal domain (373–446), made up of four helices lining the central pore (Fig. 2a). Comparison to the monomeric EscN^103–446^ crystal structure lacking the N-terminal domain^5^ reveals the most prominent structural difference at the conserved C-terminal domain, where the helices are kinked away from the ATPase domain (Fig. 2b). This difference likely stems from the helix-interrupting Val393Pro mutation, a screened functional mutant^31^ required for crystallization in the earlier study.

**Fig. 3:**
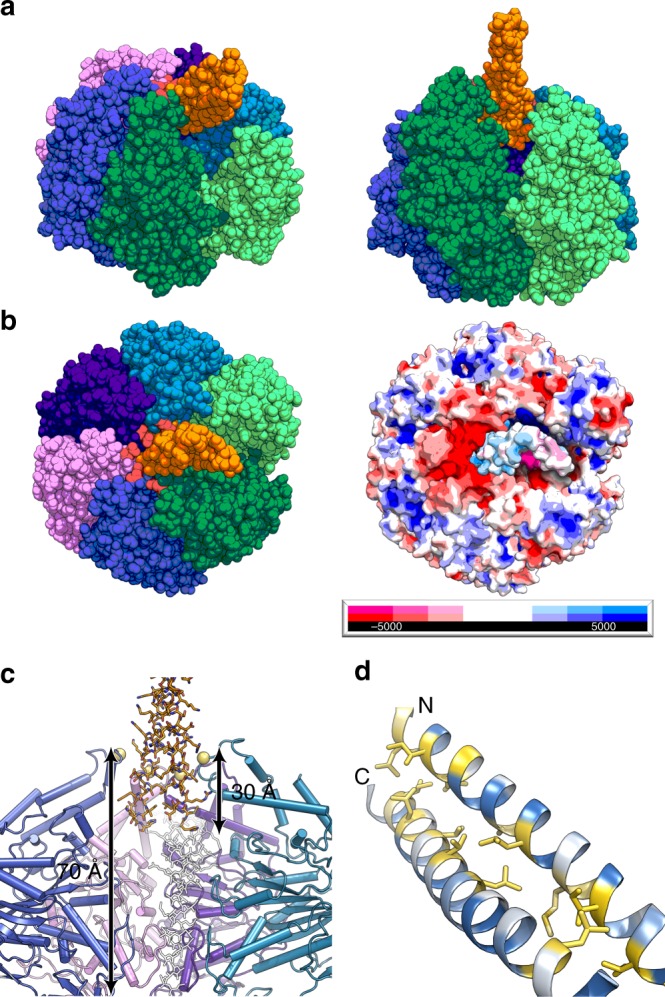
Overview of EscN-EscO complex. **a** Sphere representation of the EscN-EscO complex (class 2), coloured by subunit and shown from an angled view and side view to highlight the cleft (located between the light and dark green subunits). The EscO stalk (orange) tilts towards the cleft. **b** Top view of EscN-EscO complex, with negatively charged glutamates shown in red lining the pore, compared with the APBS-calculated electrostatic surface demonstrating the complementary charges of EscN (red, white, blue) and EscO (pink, white, light blue). **c** Stick depiction of EscO insertion into the EscN pore, where it penetrates ~30 Å; the F_1_ γ-subunit (PDB 1H8E) is overlaid in white, demonstrating its longer ~70 Å extension into the F_1_ ATPase pore. EscN Glu401 lining the pore is represented as yellow spheres. **d** EscO coloured by hydrophobicity, with hydrophobic residues coloured yellow and hydrophilic residues coloured teal; hydrophobic residues line the coiled coil interface, characteristic of this motif

The EscN hexameric structure shows marked asymmetry, with a prominent cleft between chains N_A_ and N_F_ (Figs. 2c, 3a). This hexameric arrangement has not been captured in the previous crystal structures^4,5,7,25–27^, nor accurately predicted from subsequent modelling^4,5,7,25,26^. Comparison to published catalytic heterohexamers of the F_1_- and V_1_-ATPases reveals a remarkable degree of conservation in asymmetric subunit orientation (Supplementary Fig. 5), despite their composition of alternating catalytic (β/Α) and inert (α/Β) subunits. Superposition of the EscN hexamer with the bovine F_1_-ATPase bound to transition state analogue ADP-AlF_3_ (PDB 1E1R;^30^ ~28% sequence identity with F_1_β, N_B_ Cα RMSD ~1.5 Å over 314 residues) or the *Enterococcus hirae* V_1_-ATPase in complex with AMP-PNP (PDB 3VR6^32^; ~26% sequence identity with V_1_A, N_B_ Cα RMSD ~1.3 Å over 311 residues) highlights the conserved variation in inter-subunit packing from open to tightly bound (Supplementary Fig. 5). The functional significance of EscN’s asymmetry is discussed below.

To probe where the key asymmetry-inducing conformational changes occur, careful comparison of each EscN subunit was undertaken. The N-terminal domain is largely static between subunits (Supplementary Figs 6, 7b), with approximate C6 symmetry around the pore axis (Supplementary Fig. 7a). Aligning each EscN chain by the N-terminal domain highlights a key pivot point between the N- and ATPase domains, with the ATPase and C-terminal domains essentially moving as a rigid body from subunit to subunit (Supplementary Figs. 6,  7b). Subunit N_F_, the most dynamic monomer with the least resolved density, is tilted back nearly 30° relative to the most inward angled subunit N_D_, indicating impressive motion at this pivot point (Supplementary Figs 6a, 7b).

### The EscO coiled coil interacts at the EscN C-terminal domain

EscO is the proposed EPEC homologue of the *f*T3SS central stalk protein FliJ^9^, which in turn is homologous to the central stalk proteins of the rotary ATPases^8,9,12^. Consistently, the resolved region here (EscO residues 1–30 and 92–122) forms a coiled coil similar in nature to the coiled coil region of the F_1_-ATPase γ-subunit (1E1R)^30^ and the V_1_-ATPase D subunit (3VR6)^32^, as well as to the isolated structures of T3SS central stalks FliJ (3AJW)^8^, CdsO (3K29)^10^ and YscO (4MH6);^11^ the typical coiled coil amphipathic side-chain packing is evident and supports our EscO structural model (Fig. 3d, Supplementary Fig. 4). Extending the structural and functional similarity to the rotary ATPases, both the N- and C-termini of the EscO coiled coil are observed to penetrate the EscN C-terminal pore opening (Fig. 3c, Supplementary Fig. 4). In contrast to the F_1_- and V_1_-ATPase complexes where the central stalk subunit extends ~70 Å into the catalytic core, EscO only penetrates ~30 Å, preventing interaction with all but the C-terminal domains (Fig. 3c). Previous studies on F_1_-ATPase stalks have shown that truncation of the N-terminal 50 residues^33^ or C-terminal 36 residues^34^ in the γ-subunit from thermophilic *Bacillus* only resulted in an approximate 50% decrease in torque generation. A 36-residue C-terminal γ-subunit truncation brings the end of the F_1_ rotor a similar distance into the pore as EscO, suggesting that while the increased pore entry distance by the central stalk improves torque, it is not essential for function. Isolated structures of other T3SS EscO homologues have a C-terminal helix more similar in length to the F_1_- and V_1_- ATPase components, making EscO an outlier (Supplementary Fig. 4). Counterintuitively, two studies have demonstrated that the *Salmonella* EscO homologue InvI can be tagged with an N-terminal green fluorescent protein (GFP) without impacting secretion^35,36^. Given the proximity of the EscO N terminus to the EscN hexamer cleft opening, it is plausible that the fluorescent tag (even with a relatively short linker) could reside outside the hexamer pore where it would not impede function. Alternatively, a C-terminally GFP-tagged construct of the V-ATPase-central stalk homologue Vma8p was shown to be incorporated into functional V-ATPase complexes, suggesting that GFP can be accommodated within the central cavity without disrupting function^37^.

**Fig. 4:**
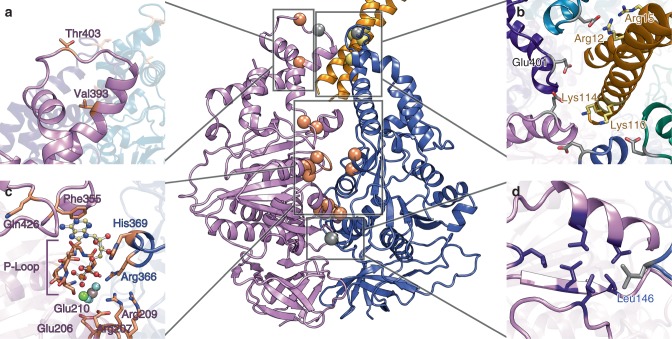
Characterized EscN/EscO mutants. Side-chain views of previously studied EscN mutants (shown in orange) mapped to the model, as well as EscN mutants (grey) and EscO mutants (yellow) characterized in this paper. The central view of the T and T′ interface provides context for the location of each site, with detailed stick views denoting side-chain locations in the **a** chaperone binding site, **b** EscN-EscO interaction interface, **c** active site and **d** hydrophobic oligomerization interface

The EscN-EscO complex structure is sufficiently resolved to characterize the binding interface between stalk and ATPase. The two resolved helices of EscO are bound in a semicircle formed by the same EscN helix-loop region (residues Leu395–Glu401) from chains N_A_ through N_E_, which congregate along one-half of the pore due to EscN’s tilt; this region in chain N_F_, the most loosely packed chain, is directly opposite the bound EscO and poorly ordered (Fig. 3b, Supplementary Fig. 8a, b). EscN creates a cradle of negative charge provided predominantly by clustered Glu401 from chains N_A_ through N_E_ (conserved in injectisome orthologues (Supplementary Fig. 9) and structurally conserved with the F_1_ β-subunit DELSEED motif), situated on the apex of the pore-proximal loop (Fig. 3b). In our model, EscO presents many electropositively charged lysines and arginines near the EscN pore entrance; specifically, Glu401 in chains A–C are positioned to interact directly with EscO Lys110 and Lys114 (Fig. 4b, Supplementary Fig. 8c). In support, mutations of either EscN Glu401 to alanine or simultaneously mutated EscO Lys110 and Lys114 to glutamate abrogated the ability of EscO to form a stable complex with EscN as measured by co-sedimentation during glycerol gradient centrifugation (Supplementary Fig. 2c–e). The EscO double lysine mutant resulted in a ~50% decrease in catalytic rate, nearing that of EscN without EscO (Supplementary Fig. 2a, b). Interestingly, Glu401Ala negatively affected the EscN ATPase rate both in the presence and absence of EscO, suggesting a cooperative role of the C-terminal domain (discussed below). In contrast, simultaneous mutation of conserved EscO Arg12 and Arg15 to glutamates (which interact with Glu401 on chain E, Fig. 4b) had no significant effect on either complex formation or catalytic activity compared to wild-type EscN-EscO (Supplementary Fig. 2a, b, d–e), indicating that their position is less important for the EscO-EscN interaction and function. In addition to these electrostatic interactions, conserved residues Leu396 and Ile399 (Supplementary Fig. 9) contribute to a hydrophobic collar below the Glu401 ring. These residues form close interactions with hydrophobic residues on EscO (Supplementary Fig. 8d), and may have a similar rotation-facilitating role as in F_1_-ATPases, where the homologous region was termed a molecular bearing^38^.

### Active site architecture and implications for catalysis

**Fig. 5:**
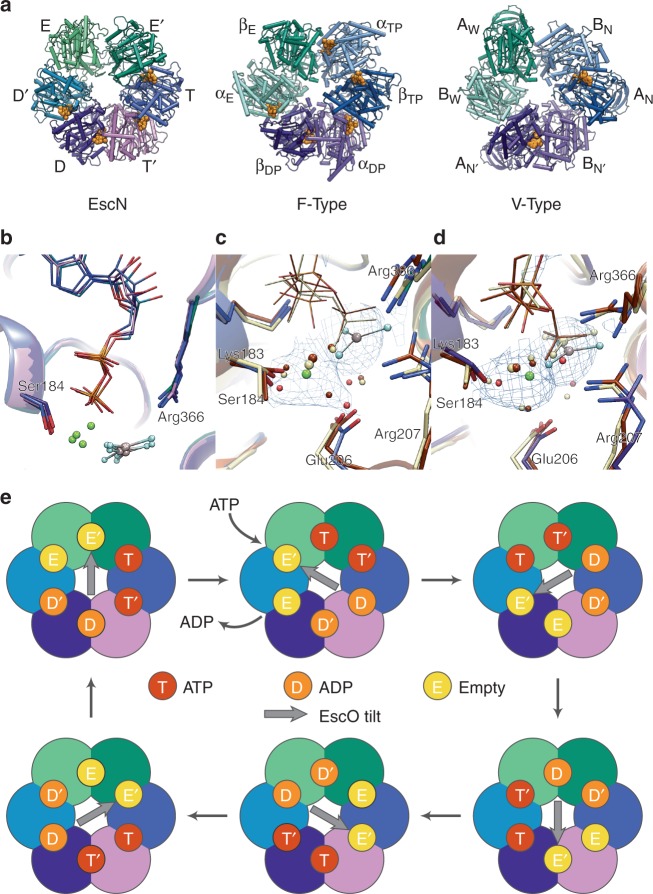
EscN catalysis. **a** Top view of EscN homohexamer without EscO compared to F_1_ (1E1R) and V_1_ (3VR6) heterohexamers, with subunit names labelled along the periphery; nucleotides are shown as orange spheres. **b** Comparison of the EscN inter-site distances for all four nucleotide-bound sites, with Cα distances between Ser184 and Arg366 progressively decreasing from 11.9, 11.2, 11.1, and 10.8 Å through sites T to D′. Overlay of EscN (subunits coloured as in (A)), F_1_-ATPase (coloured tan, 1E1R) and V_1_-ATPase (coloured brown, 3VR6). **c** T, β_TP_ and A_N_ sites and **d** D, β_DP_ and A_N′_ sites, demonstrating the similarities in side-chain residues and water positions. Blue density is carved around Mg^2+^ and waters from a class 2 map (*B*-factor sharpened by a factor of −150 and contoured at 10*σ*), showing the broad density that encompasses the coordinating waters. **e** Possible rotational catalysis mechanism of EscN and EscO, with the central arrow representing the direction of EscO’s tilt

The resolution of our EscN reconstructions has permitted detailed modelling of the active sites, including bound substrates and conserved active site waters (Figs. 1c, 4c–d, Supplementary Fig. 10) that reveal strong parallels with the F_1_- and V_1_- ATPase active site architectures. The EscN active site location at the subunit interface and key motifs are conserved with the latter, including the P-loop (residues 179–185), catalytic glutamate (Glu206), and arginine finger (Arg366) (Fig. 4c, Supplementary Fig. 9). Clear density is observed for the transition state analogue Mg^2+^ADP-AlF_3_ in four of the six active sites, with the most detailed density observed for the chain N_B_ active site at the interface with chain N_A_. EscN’s ADP nucleotide is stabilized in the binding pocket by an aromatic stacking interaction with Phe355 (conserved in F_1_-ATPases (bovine βTyr345)^30^ and V_1_-ATPases (*E. hirae* αPhe425))^32^, which accesses the site through a turn flanked by conserved His354 and Pro356. However, EscN lacks a large loop found in F_1_-ATPases (bovine β419–427) and a similarly placed bulky phenylalanine in V_1_-ATPases (*E. hirae* αPhe506) that stabilize the nucleotide ribose moiety. A looser nucleotide interface could contribute to EscN’s observed nucleotide-dependent oligomerization, and may relate to the recycling of the T3S ATPase complex, which has been shown to be in dynamic exchange between a membrane-associated hexameric assembly and a cytoplasmic hetero-trimeric complex with the peripheral stalk component^39^. The P-loop stabilizes the α- and β-phosphates; multiple backbone amides point towards the β-phosphate, with conserved Gly182 facilitating the required tight loop conformation. Further, the planar AlF_3_ aligns well with the position of the γ-phosphate (or analogues) in several bovine F_1_ β_TP_ sites (PDB 1E1R^30^, 1H8E^40^) and in the *E. hirae* V_1_-ATPase (3VR6) (Fig. 5c–d). Coordination is by three conserved, electropositive residues with similar conformations amongst homologues: Lys183 from the P-loop, Arg207 and the arginine finger Arg366 from the preceding chain, essential for oligomerization (Fig. 5c–d). The catalytic magnesium ion is coordinated by the side-chain hydroxyl of Ser184 (homologous to the conserved P-loop threonine in F_1_ and V_1_-ATPases), one fluoride from AlF_3_ and one β-phosphate oxygen. In ATP synthase, the remaining hexadentate coordination comes from three water molecules; variable *B*-factor sharpening here reveals additional density peaks around the magnesium ion, consistent with the coordinating waters that we have modelled (Fig. 5c–d, Supplementary Fig. 10), see below. Density for more active site waters is also observed at positions conserved with the F_1_ active site, notably the key water bridging the catalytic Glu206 and the AlF_3_ (Fig. 5c–d, Supplementary Fig. 10). Our data is in keeping with the classic catalytic ATPase mechanism, where Glu206 activates the catalytic water for nucleophilic attack on the ATP γ-phosphate, while the Mg^2+^ and γ-phosphate-coordinating residues (Lys183, Arg207 and Arg366) draw electron density from the phosphorous atom to render it a more attractive electrophile (Fig. 1c).

The assembled EscN-EscO structure determined here allows atomic level understanding of its similarities to and customized differences with the F_1_/V_1_-ATPases. Foremost, the different conformational states at each EscN active site mirror what has been observed in EscN’s rotary relatives. Although EscN’s homohexameric complex distinguishes it from the alternating inactive (α/B) and active (β/A) subunits of F_1_ and V_1_, clear conformational parallels are present. Historically, the three catalytic states in F_1_ are termed the empty state (β_E_), the ATP-bound state (β_TP_) and the ADP-bound state (β_DP_) based on the first structure with identifiable bound substrates^38^. V_1_ has analogous sites, described as wide-open (A_W_) and narrowly closed (A_N_ and A_N′_)^41^. The empty site (β_E_ or A_W_) allows nucleotide exchange and transitions to the first nucleotide-bound site upon ATP binding (β_TP_ or A_N_), where the inter-subunit distance is tighter and the C-terminal domain (lever domain) moves from open to closed^42^. From there, the subunits show subtle active site repositioning as they shift to the proceeding β_DP_ or A_N′_ state, thought to reflect the ATP hydrolysis-competent conformation^32,43^. The cycling between these sites has been shown to generate torque in F_1_- and V_1_-ATPases.

We observe a series of conformational changes between EscN’s subunits and active sites consistent with the observed catalytic states of F_1_/V_1_-ATPases (Supplementary Figure 7c, d). Based on the EscN active site features and supported by similarities to the F_1_/V_1_-ATPase active sites discussed below, we will refer to the active site states as E (empty; equivalent to the F_1_ β_E_ and V_1_ A_W_ states), E′ (F_1_ α_TP_, V_1_ B_N_), T (analogous to ATP-bound state; F_1_ β_TP_, V_1_ A_N_), T′ (F_1_ α_DP_, V_1_ B_N′_), D (analogous to ADP-bound state; F_1_ β_DP_, V_1_ A_N′_) and D′ (F_1_ α_E_, V_1_ B_W_) (Fig. 5a). The EscN E state subunit is tilted furthest back from the pore and is the least well resolved, superposing with the F_1_ β_E_ and V_1_ A_W_ states (2.3 and 1.5 Å Cα RMSD, respectively; Supplementary Fig. 7c, d) and displaying no clear density for bound substrates. Moving clockwise, the EscN E′ active site is positioned at the solvent-exposed cleft and is unoccupied by substrate, corresponding to the inactive F_1_ α_TP_ and V_1_ B_N_ sites (between the catalytically active empty and nucleotide-bound states). The E′ state subunit conformation and active site accessibility is well placed for ATP binding, which would trigger the associated conformational changes. The subsequent four EscN sites show the tightest subunit interfaces, with prominent densities for ADP, Mg^2+^ and AlF_3_ (Fig. 5a); we propose that these sites represent the progression from ATP binding, through hydrolysis and eventually to the ADP-bound state. The interface area at each site gradually increases from the T site through the D′ site, indicating a gradual tightening through the catalytic cycle. In both the F_1_ and V_1_ structures, the transitions from the β_TP_/A_N_ to β_DP_/A_N′_ states are characterized by a tighter subunit interface and accompanying shift of the catalytically critical arginine finger located on the neighbouring subunit; this arginine is thought to be involved in stabilizing the transiently formed pentacoordinated state of the γ-phosphate^44^. Comparison of the EscN active sites with bound substrates reveals a similar pattern, with the distance between Arg366 guanidinium carbon and the aluminium atom of AlF_3_ decreasing by ~0.5 Å between the T and D active sites (Fig. 5b). At this resolution, we observe the active site Mg^2+^ to have pentadentate coordination with two water molecule ligands compared to the more typical hexadentate coordination that is observed in the F_1_ and V_1_ structures; this is consistent with the only published T3SS ATPase with a defined active site Mg^2+^ coordination (*Shigella flexneri* Spa47^84–430^ PDB 5YBI^27^). In EscN’s T site, the Mg^2+^ coordination shows clear trigonal bipyramidal geometry, with the water positions incrementally shifting to adopt a square pyramidal geometry in the D site (Supplementary Fig. 10). Combining these details, we hypothesize that the T site (Fig. 5c), having the loosest interface, houses freshly bound ATP; the D site (Fig. 5d), showing the most similarity to the β_DP_/A_N′_ sites, represents the catalytic conformation^43^. Sites T′ and D′, unique to homohexameric rotary ATPases, represent accordingly the intermediate transitions between binding, hydrolysis and ADP and/or phosphate release (Fig. 5e).

## Discussion

The structure of the EscN homohexamer in complex with substrate analogue and inner stalk EscO represents a significant advance over previously available structures of monomeric orthologues^4,5,7,25–27^, which lacked the varied conformational states observed here and the interface with the inner stalk. The remarkable conservation of sequential active site states from F_1_/V_1_-ATPase heterohexamers provides direct atomic evidence of a related torque-generating catalytic cycle. A role for rotation of the *f*T3SS central stalk FliJ in flagellar assembly has been previously hypothesized but not directly shown, and remained a conundrum given the homohexameric nature. The *v*T3SS EscO stalk characterized here has been shown to complement the *f*T3SS homologue FliJ in vivo, upregulating ATPase activity of the *f*T3SS FliI and partially rescuing motility of a *fliJ* deletion mutant^9^. FliJ, in turn, has been shown to bind and upregulate a V_1_-ATPase catalytic A_3_B_3_ heterohexamer, and can be rotated in an imperfect manner (likely a result of its lower affinity binding)^12^. We propose that the EscN-EscO atomic structures presented here are therefore generally representative of the flagellar and virulence T3S ATPase variants, and directly support a role of torque generation and subsequent rotation of the central stalk in the process of T3SS.

In the F_1_- and V_1_-ATPases, the C-terminal helical lever domain is important for torque generation. ATP binding, hydrolysis and release serve to induce conformational changes within this domain that provide the force to rotate the central stalk^42^, with hydrolysis of three ATP molecules resulting in one full rotation. The structure presented here shows that EscN can adopt similar C-terminal domain conformations; the lever is closed in nucleotide-bound sites T through D′, and open in nucleotide-free sites E and E′. Large-scale conformational changes occur upon nucleotide binding (E′ to T; closing toward the pore) and release (D′ to E; opening away from the pore), fuelled by movement between EscN’s N-terminal and ATPase domains (Supplementary Figs 6a, 8b). These alternating conformations create a rippling dynamic as the subunits undulate in and out during ATP hydrolysis (Supplementary Movie 1). By analogy to the F_1_/V_1_-ATPases, this could be translated into torque on the EscO stalk delivered by the C-terminal helical domain. As EscN’s subunits are pushed towards and away from the pore during a rotary cycle, the electronegative pockets we observe formed by successive Glu401s would circle around one-half of the pore (Fig. 3b); residues along the EscO stalk, particularly Lys110 and Lys114 (the former conserved with *v*T3SS central stalks^9^), likely follow these glutamates via complimentary electrostatics, causing it to rotate (Supplementary Fig. 8c). Simultaneously, hydrophobic interactions from the Leu396 and Ile399 molecular bearing^38^ help to secure EscO within the pore while permitting smooth rotation (Supplementary Fig. 8d). We hypothesize this interaction efficiently translates the torque from EscN’s conformational changes into a rotational force on EscO. Our structural data provides the foundation to probe other customized differences that may be unique to the homohexameric T3SS catalytic cycle compared to the well-studied F- and V-type ATPases. The presence of six (presumably active) catalytic sites suggests that EscN is less efficient than the latter, requiring hydrolysis of six molecules of ATP rather than three for a full 360° rotation. Future biophysical experiments will be needed to clarify how much torque is generated per ATP, and to identify if and where energy is being lost in this presumably more primitive homohexameric motor.

The precise role(s) played by the ATPase in assembly and function of the T3S injectisome and flagellum remain poorly understood. At the heart of both nanomachines, the conserved T3SS export apparatus consists of a membrane-embedded export apparatus (EscRSTUV or FliPQR, FlhB and FlhA, respectively) and the cytoplasmic ATPase complex. T3S requires both ATP hydrolysis and the movement of protons across the cytoplasmic membrane by the export gate for efficient secretion^19,45^. PMF is the prominent energy source, with disruption by the proton ionophore CCCP inhibiting secretion^16^. The export gate EscV (flagellar FlhA) is composed of a nonameric cytoplasmic domain and a structurally uncharacterized transmembrane region proposed to be a potential proton channel and function as a proton/protein antiporter^46^. Export gate FlhA has been shown to be capable of translocating protons and sodium ions^46^ and supports secretion even in the absence of the ATPase in certain mutant backgrounds^47^. The ATPase, however, improves the efficiency of this process, with interaction of the ATPase complex required to fully activate the export gate^18^. These studies point to a PMF-dependent cooperative mechanism for T3S energetics involving both the export gate and the ATPase complex.

In keeping, direct interactions between the ATPase complex and export gate have been documented. Data from both the *v*T3SS^48^ and the *f*T3SS^18^ suggest that the central stalk protein directly interacts with the major export gate protein EscV/FlhA^49^, which could draw the ATPase-central stalk complex within interacting distance of the gate. Specifically, the central stalk FliJ in complex with ATPase FliI and peripheral stalk FliH interact with FlhA through conserved Phe72 and Leu76^50^, while mutants mapping to the same region of *Pseudomonas aeruginosa* central stalk PscO upregulated secretion via a suggested interaction with the export gate PcrD^48^. For both, it was hypothesized that the interaction increased the efficiency of substrate secretion by modulating the efficiency with which the T3SS uses the PMF. The region implicated in the interaction of the central stalk and export gate is not resolved in our structure, although FliJ residues Phe72 and Leu76 are conserved in EscO (Tyr51 and Leu55, Supplementary Fig. 4). Assuming a continuous coiled coil motif in keeping with the characteristic primary sequence, these residues would be positioned approximately at the apex of the EscO structure, ideal for interaction with the export gate. Such an interaction could allow EscO to affect the conformational state of EscV, or to facilitate passage of substrates from EscN to EscV. Remarkably, recent experiments have shown that the ATPase is capable of energizing secretion even in the absence of bulk PMF;^51^ based on this observation, it was proposed that the ATPase complex may itself be involved in the generation of a local proton gradient that is used by the export gate. Our structure of the EscN-EscO complex and its extensive structural homology with the F_1_/V_1_-ATPases (which can fuel outward pumping of protons) supports such a hypothesis. These similarities raise the intriguing possibility that elements of the export gate might function in a manner similar to the F_o_/V_o_ components, acting as a channel through which protons are pumped across the inner membrane.

**Fig. 6:**
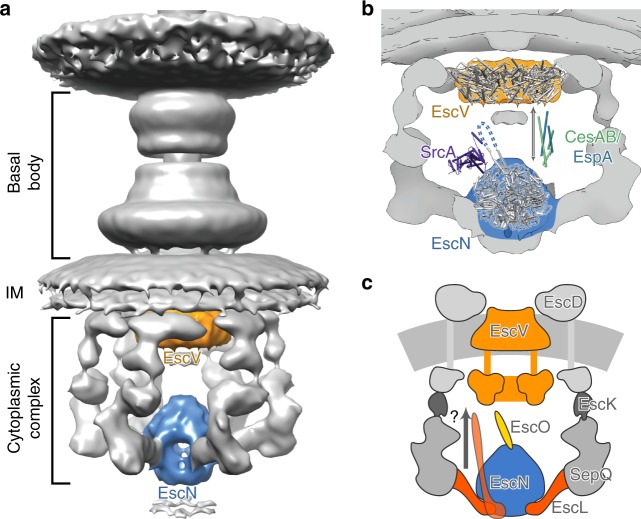
Function of EscN-EscO in the injectisome. **a** Overview of EscN and EscV location in the context of the T3SS nanomachine (based on in situ tomography of the *Salmonella* injectisome, EMDB 8544). **b** Putative binding sites of type III chaperone-substrate complexes, based on previous studies on SrcA/SsaN and CesA/EspA/EscV. Each binding site brings the cargo into the lumen between EscN and EscV. The unresolved region of EscO is represented by a dotted line. **c** Schematic of proteins present in the cytoplasmic subcomplex. It has been hypothesized that the entire ATPase complex may rise towards the inner membrane to interact with the export gate

A further role of the T3S ATPase in substrate targeting and unfolding has been suggested from prior work. Most T3S effector substrates are localized to the T3S apparatus in a partially unwound state, a result of complex formation with specialized chaperones^23^. Studies have demonstrated that some of these chaperone-effector complexes can both bind to the T3S ATPase and be dissociated and unfolded in an ATP-dependent manner^20–22^ (typically mediated by the chaperone whether alone^25,52^ or in complex with effector^52,53^, although interaction of the effector signal sequence with the ATPase has also been demonstrated^54,55^). Published mutations mapping to the EscN C-terminal domain in our structures here have suggested an important role in substrate recognition^5,20,25,56^. Specifically, Allison et al.^25^ identified mutants in a conserved region of the C-terminal helical domain of *Salmonella* *typhimurium* SPI-2 T3S ATPase SsaN that abrogated interaction with the multicargo chaperone SrcA^25^ (Figs. 4a, 6c). Our structures of the EscN hexamer and its varying catalytic states provides a potential structural basis by which ATP hydrolysis induced effector–chaperone dissociation can be achieved. As discussed above, we observe a significant degree of conformational change in the C-terminal domain throughout the catalytic cycle detailed by our structure and we propose that these, especially the transition to the empty (E, E′) states, will disrupt chaperone binding to the effector and/or ATPase C-terminal domain. In the case of EPEC CesAB-EspA, Chen et al.^53^ found that it bound only to oligomerized EscN (with deletion of EscN’s N-terminal oligomerization domain abrogating the interaction), suggesting that some chaperones may selectively bind subunit conformations only adopted in the assembled state. The function of the central stalk in this is not clear; interactions of specific chaperones and central stalk homologues have been demonstrated^57,58^, and Allison et al.^25^ identified the SrcA binding site based on its structural similarity to the globular portion of the F_1_-ATPase-central stalk γ-subunit (absent in the coiled coil structure of EscO and other T3S homologues; Supplementary Fig. 4). For the studies with *v*T3SS ATPase homologues demonstrating chaperone-effector dissociation^20–22^, the central stalk component was not included, suggesting that the ATP hydrolysis-induced conformational changes alone are sufficient. Stabilization of the hexamer by the central stalk could potentiate this or, given the proposed role in activation of the export gate discussed above, the central stalk could facilitate communication between the ATPase and export gate upon effector release (Fig. 6b). Indeed, some chaperone-effector complexes have been shown to bind both the ATPase and the export gate including *v*T3SS CesAB (chaperone)-EspA (EPEC filament)^53,59^ and *f*T3SS FliT (chaperone)-FliD (flagellar filament cap)^56,60^, suggesting a cooperative role in chaperone recruitment, targeting and secretion regulation (Fig. 6). FliT-FliD in particular has been shown to bind to monomeric *f*T3SS ATPase FliI in the presence of peripheral stalk FliH, a complex hypothesized to facilitate escort of the effector to the export gate^56^. In keeping with this, a recent study has demonstrated that *f*T3SS ATPase subunits are in a continual state of exchange between membrane-associated and cytoplasmic complexes^39^. As discussed above, the looser inter-subunit active site packing and shallower central stalk binding of the EscN-EscO complex compared with F/V-ATPases could allow for readier subunit dissociation to facilitate this exchange. Finally, we note that the structures of both CesAB-EspA and FliT-FliD complexes have coiled coil structures with similar characteristics to the central stalk components (PDBs 1XOU^61^ and 6CH2^60^, Supplementary Fig. 4) and it is plausible that some chaperones may interact with the ATPase in a manner related to the observed EscO interaction here, potentially providing an additional path for subsequent targeting to the export gate. The latter possibility is intriguing given the breadth of apparatus and effector substrates requiring passage in the various T3SS species.

In summary, we present the atomic details of an assembled T3SS ATPase in complex with its central stalk protein. The structure provides a molecular foundation for the differential rotational states of the homohexamer, for chaperone/substrate recognition and for subsequent ATPase-driven secretion initiation. In turn, we can begin to probe deeper into the underlying biophysics of T3SS ATPase torque generation and rotational mechanism and how this ancient protein family has been preserved through its cooperation with the T3SS in widespread bacterial pathogenicity.

## Methods

### Expression and purification of EscN^29–446^ and EscO^1–125^

The genes for EscN and EscO were cloned from EPEC genomic DNA (*E. coli* O127:H6 E2348/69)^62–64^ into individual pET28a vectors, each with a thrombin-cleavable N-terminal 6His tag (for primer list, see Supplementary Table 1). The first 28 residues were omitted from EscN, as this improved expression and stability; EscO was expressed as a full-length protein. The vectors pET28^EscN-6NHis^ and pET28^EscO-6NHis^ were transformed separately into *E. coli* BL21 (λDE3, Sigma) and expressed in 2 L and 0.5 L ZYP-5052 auto-induction media, respectively (grown at 37 °C for 3.5 h followed by 22 °C for 16 h). Cells were harvested by centrifugation at 6200 × *g* for 20 min, yielding approximately 25 g pET28^EscN-6NHis^-transformed cells and 7 g pET28^EscO-6NHis^-transformed cells. Pellets were resuspended in lysis the buffer (20 mM HEPES, pH 7.5, 500 mM NaCl, 5% glycerol, 0.5 mM Tris(2-carboxyethyl)phosphine hydrochloride (TCEP-HCl), 15 mM imidazole, EDTA-free protease inhibitor tablet (Roche)) at a ratio of 4 mL buffer to 1 g cell pellet. The cells were lysed by two passes through an EmulsiFlex-C5 homogenizer (Avestin), and insoluble material from the lysate was removed by centrifugation at 40,000 × *g* for 30 min. The lysate was filtered and passed over a 5 mL HisTrap HP (GE Healthcare) in the case of EscN, and over a 1 mL HisTrap HP (GE Healthcare) for EscO. The columns were washed with 20 column volumes (CV) of 50 mM imidazole wash buffer (20 mM HEPES, pH 7.5, 500 mM NaCl, 5% glycerol, 0.5 mM TCEP-HCl, 50 mM imidazole), followed by 5 CV of 75 mM imidazole wash buffer and 5 CV 100 mM imidazole wash buffer; protein was eluted in 300 mM imidazole buffer. To remove the his-tag, the proteins were desalted into EscN-EscO buffer (20 mM HEPES, pH 7.5, 500 mM NaCl, 5% glycerol, 0.5 mM TCEP-HCl) and incubated with bovine α-thrombin (Haematologic Technologies Incorporated) at 4 °C overnight at a molar ratio of 1:1000. Imidazole stock was added to the proteins to a final concentration of 15 mM, and the proteins were passed once more over a HisTrap HP (GE Healthcare) column to remove any uncleaved sample. The proteins were once again desalted into EscN-EscO buffer using a PD-10 desalting column (GE Healthcare), and concentrated with Amicon Ultra centrifugal filters (EMD Millipore) to a final concentration of ~25 mg/mL for EscN^29–446^ and ~5 mg/mL for EscO^1–125^. Mutants of EscN^29–446^ and EscO^1–125^ were made using the QuikChange mutagenesis kit (Strategene), and purified using the same protocol (Supplementary Table 1).

### Glycerol gradient centrifugation of EscN-EscO complex

EscN^29–446^ and EscO^1–125^ were incubated together at 4 °C for a minimum of 8 h at 5.0 and 0.8 mg/mL, respectively (1:2 molar ratio EscO:EscN, 3-fold excess EscO from the expected 1:6 stoichiometry), in EscN-EscO buffer containing aluminium fluoride ATP hydrolysis transition state analogue (EscN-EscO-ADP-AlF_3_: 20 mM HEPES, pH 7.5, 500 mM NaCl, 5% glycerol, 0.5 mM TCEP-HCl, 1 mM ADP, 3 mM MgCl_2_, 6.25 mM KF, 1.25 mM AlCl_3_). A 10–25% glycerol gradient was made with EscN-EscO-ADP-AlF_3_ buffer using a Gradient Station (BioComp Instruments), and allowed to cool for 30 min at 4 °C. The EscN-EscO mixture (200 µL) was added to the top of the gradient and centrifuged at 367,600 × *g* at 4 °C for 5 h in a SW 55 Ti rotor (Beckman Coulter). The gradient was fractionated into 14 fractions using the Piston Gradient Fractionator (BioComp Instruments), and protein was visualized with the Triax UV detector (BioComp Instruments) and by sodium dodecyl sulphate-polyacrylamide gel electrophoresis. Fractions containing the assembled protein complex (approximately halfway down the gradient) were combined and desalted into cryo-EM buffer (20 mM HEPES, pH 7.5, 500 mM NaCl, 2% glycerol, 0.5 mM TCEP-HCl). The desalted protein complex was concentrated to ~2 mg/mL in Amicon Ultra centrifugal filters (EMD Millipore).

For analytical gradient centrifugation runs, the same protocol was used but with EscN at 2.0 mg/mL, and EscO at 0.7 mg/mL.

### Cryo-EM reconstruction of the EscN-EscO complex

Aliquots of 3 μL sample of the EscN-EscO complex were applied to glow-discharged (60 s on carbon side) Quantifoil grids (Copper, 300 mesh, R1.2/1.3). The grids were blotted for 3 s at 100% humidity and plunge-frozen into liquid ethane using a Vitrobot Mark IV. Grids were imaged on a 300 kV Titan Krios cryo-EM equipped with Gatan K2 Summit direct electron detector. Images were taken on the K2 camera in dose-fractionation mode at a calibrated magnification of 49,020, corresponding to 1.02 Å per physical pixel (0.51 Å per super-resolution pixel). The dose rate on the specimen was set to be 9.61 electrons/Å^2^/s and total exposure time was 6 s, resulting in a total dose of 57.67 electrons/Å^2^. With dose-fractionation set at 0.2 s/frame, each movie series contained 30 frames and each frame received a dose of 1.92 electrons/Å^2^. Fully automated data collection was carried out using SerialEM with a nominal defocus range set from −1.2 to −2.5 μm^65^.

A total of 2645 movies were collected at super-resolution (0.51 Å/pixel). Initial processing was carried out in Relion3^66^ using MotionCor2^67^ to bin (1.02 Å per pixel after binning) and align the 30 frames, and sum to a single micrograph with dose filtering; and CTFFIND4 to determine the contrast transfer functions^68^. Using Relion3, ~600,000 particles were automatically picked, reference-free 2D classification was performed and ~198,000 particles were kept in the good class averages. An ab initio model was generated in cryoSPARC v2^69^. Relion3 was used for 3D classification without imposing any symmetry, which generated one good 3D map out of four with ~110,000 particles. Within this good class, a second round of 3D classification was done in Relion3 that generated two distinct classes (class 1 with ~58,000 particles and 2 with ~55,000). 3D auto-refinements for classes 1 and 2 were subsequently done in Relion3 followed by contrast transfer function refinement and Bayesian Polishing^70^. Semi-automated post-processing of the refined maps, including automated soft masking, modulation transfer function and *B*-factor sharpening, were performed in Relion3 and yielded the final maps. Fourier shell correlation at a criteria of 0.143 reported a 3.34 Å resolution for class 1 map and a 3.29 Å resolution for class 2 map, both using gold-standard refinement procedures and high-resolution noise substitution to correct soft-mask effects^71^.

### Model building into cryo-EM maps

To build EscN^29–446^ into the class 1 reconstruction (3.34 Å resolution), an initial model based on the crystal structure of EscN^103–446^ (2OBM)^6^ was used in combination with a Phyre2^72^ homology model of the N-terminal domain (residues 29–102) from flagellar homologue FliI (5B0O)^7^. These models were first rigid-body fit into the map using Chimera^73^, and then refined with phenix.real_space_refine^74^. Iterations of manual refinement using Coot^75^ were followed by runs of phenix.real_space_refine and phenix.refine. Ligands were fit into the active sites and key interactions were linked using phenix.link_edits.

The model of EscN^29–446^ from the class 1 map was then refined into the class 2 map (3.29 Å resolution, with density of ~60 residues from EscO^1–125^) using phenix.real_space_refine. Two poly-alanine helices were then fit into the density of EscO using Coot, with their directionalities clearly visible based on the orientation of residue Cβs. The side-chain density, especially for the regions extending into the EscN hexamer, was of sufficient quality to allow confident sequence assignment for residues 0 (remaining post-cleavage affinity tag residue)–30 on helix 1 and 92–122 on helix 2.

Structural figures were made using PyMOL^76^ and Chimera^73^, and the ABPS plugin^77^ for electrostatic surface representation.

### EnzChek ATPase activity assays

Steady-state EscN ATPase activity was assayed using the EnzChek phosphate assay kit (Thermo Scientific). EscN^29–446^ and EscO^1–125^ (and mutants thereof) were incubated overnight in EscN-EscO buffer at a 1:1 molar ratio (25 µM each) at 4 °C to induce complex formation. The EscN-EscO incubation was performed in the presence of ADP-AlF_3_ (1 mM ADP, 3 mM MgCl_2_, 6.25 mM KF, 1.25 mM AlCl_3_) to improve EscN^29–446^ hexamerization unless otherwise specified; due to the inhibitory nature of ADP-AlF_3_ combined with its requirement for complex stabilization, it was diluted out of the enzyme mixture immediately prior to the beginning of the assay (50× diluted in the final reaction mixture). All assays were performed at 37 °C at 3 mM ATP with 500 nM EscN (following EnzChek protocol), in 24 µL reactions in 384-well plates (Corning 3540), using a Synergy H4 Microplate Reader (BioTek Instruments). Absorption reads at 360 nm were taken at 10 s intervals, and a linear portion over a 1 min timespan was used to calculate relative steady-state reaction rate.

### Reporting summary

Further information on experimental design is available in the Nature Research Reporting Summary linked to this article.

## Supplementary information

Supplementary Movie 1

Supplementary Information

Reporting Summary

Description of Additional Supplementary Files

## Data Availability

Data supporting the findings of this manuscript are available from the corresponding authors upon reasonable request. A reporting summary for this Article is available as a Supplementary Information file. Cryo-EM maps and atomic coordinates have been deposited to the EMDB and PDB with accession codes EMD-9390, EMD-9391, PDB ID 6NJO, and PDB ID 6NJP.
