# Supplementary material for: Cryo-EM structure of the homohexameric T3SS ATPase-central stalk complex reveals rotary ATPase-like asymmetry
Source: Nat Commun. 2019 Feb 7;10:626. doi: 10.1038/s41467-019-08477-7 (PMC6367419; doi:10.1038/s41467-019-08477-7)
Supplement: Supplementary file 2 — Supplementary Information [file 41467_2019_8477_MOESM2_ESM.pdf]

SUPPLEMENTARY INFORMATION

Cryo-EM structure of the homohexameric T3SS ATPase-central stalk complex reveals rotary ATPase-like asymmetry

Majewski *et al*.

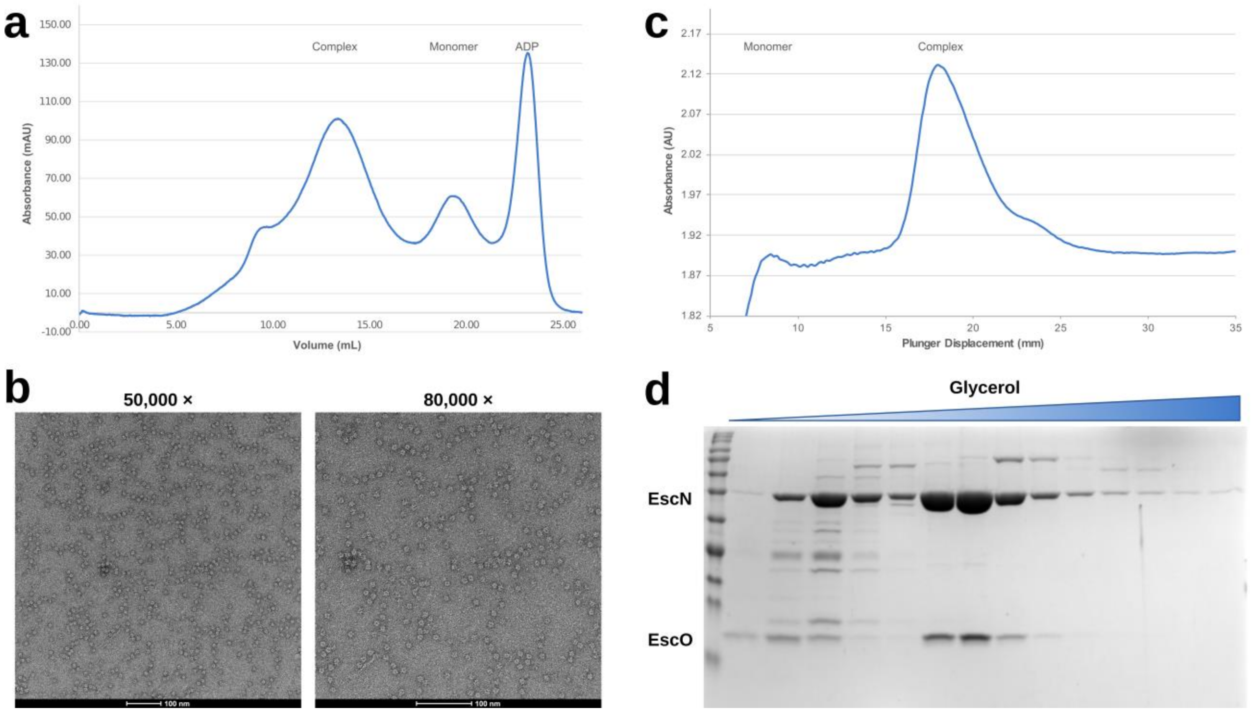

Supplementary Figure 1: EscN-EscO complex purification. (a) Size-exclusion UV chromatogram of the EscN-EscO complex using a Superose 6 10/300 column (GE Healthcare), with EscN-EscO predominantly in an oligomeric population. 500 µL of EscN (10 mg/mL) EscO (1 mg/mL) mixture were injected. **(b)** Negative-stain micrographs of the EscN-EscO complex with clear ~10 nm rings. **(c)** UV chromatogram and **(d)** SDS-PAGE of 10-25% glycerol gradient purification, showing EscO co-sedimenting with EscN in a large oligomeric population at high purity. A 200 µL mixture of EscN (2.0 mg/mL) and EscO (0.7 mg/mL) were loaded onto the gradient.

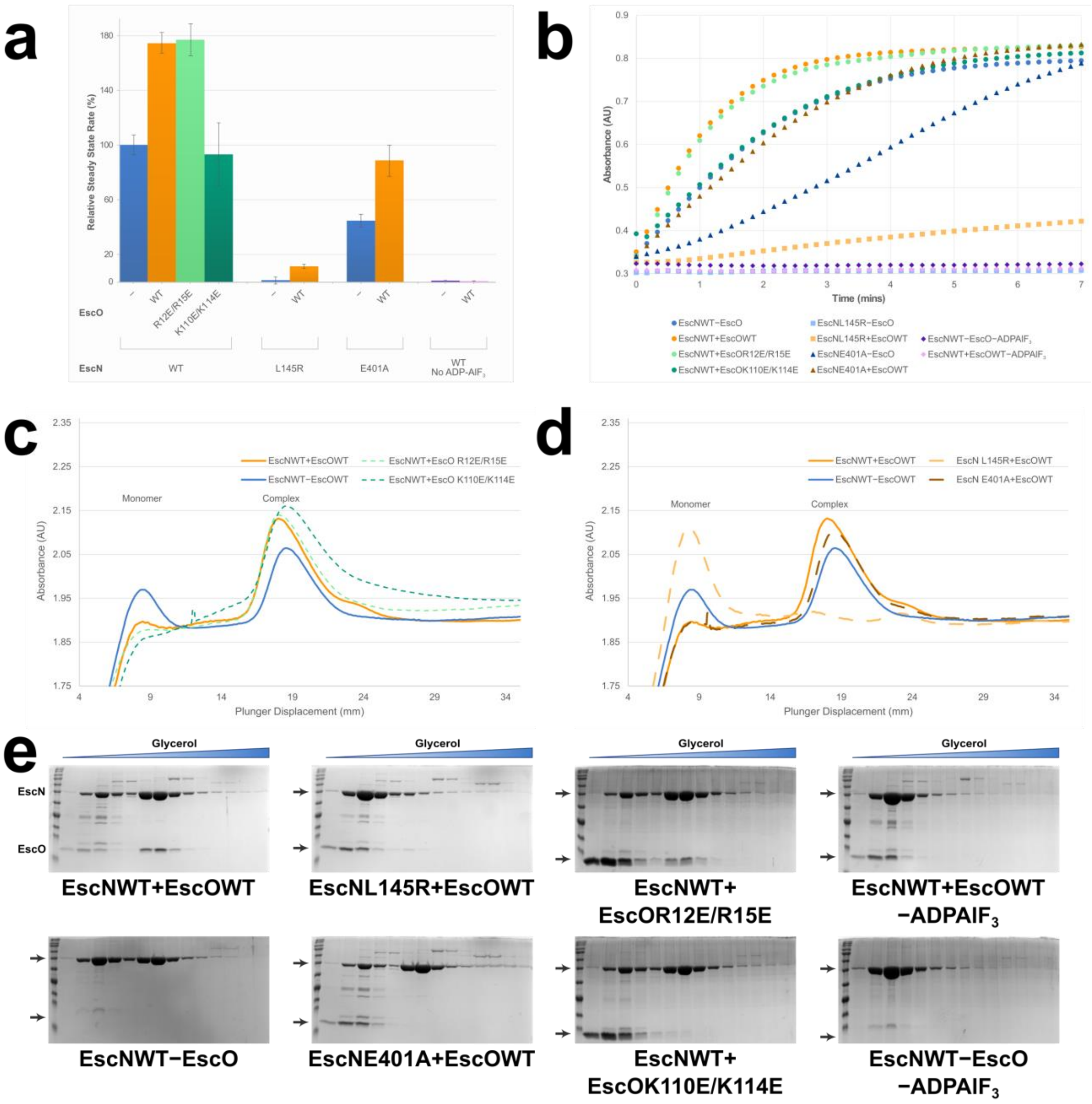

Supplementary Figure 2: Kinetics and glycerol gradient analysis. Steady-state kinetic analysis of EscN-EscO rate assayed by EnzChek phosphate detection assay, shown as a **(a)** bar graph of steady-state slope taken over a linear section of one minute (error bars show standard deviation from n=4 slopes), and **(b)** A_{360} time-course showing EnzChek product formation over time. Both plots are averaged from four replicates. **(c,d)** Glycerol gradient UV chromatograms of EscN-EscO complex, showing monomeric vs oligomerized populations for various mutants. A 200 µL mixture of EscN (2.0 mg/mL) and/or EscO (0.7 mg/mL) were loaded onto the gradient. **(e)** Gels of glycerol gradient purifications, highlighting the oligomerization incompetence of EscN L145R and EscN lacking $ADPAlF_3$, and the destabilization of the EscN-EscO interface in EscN E401A and EscO K110E/K114E.

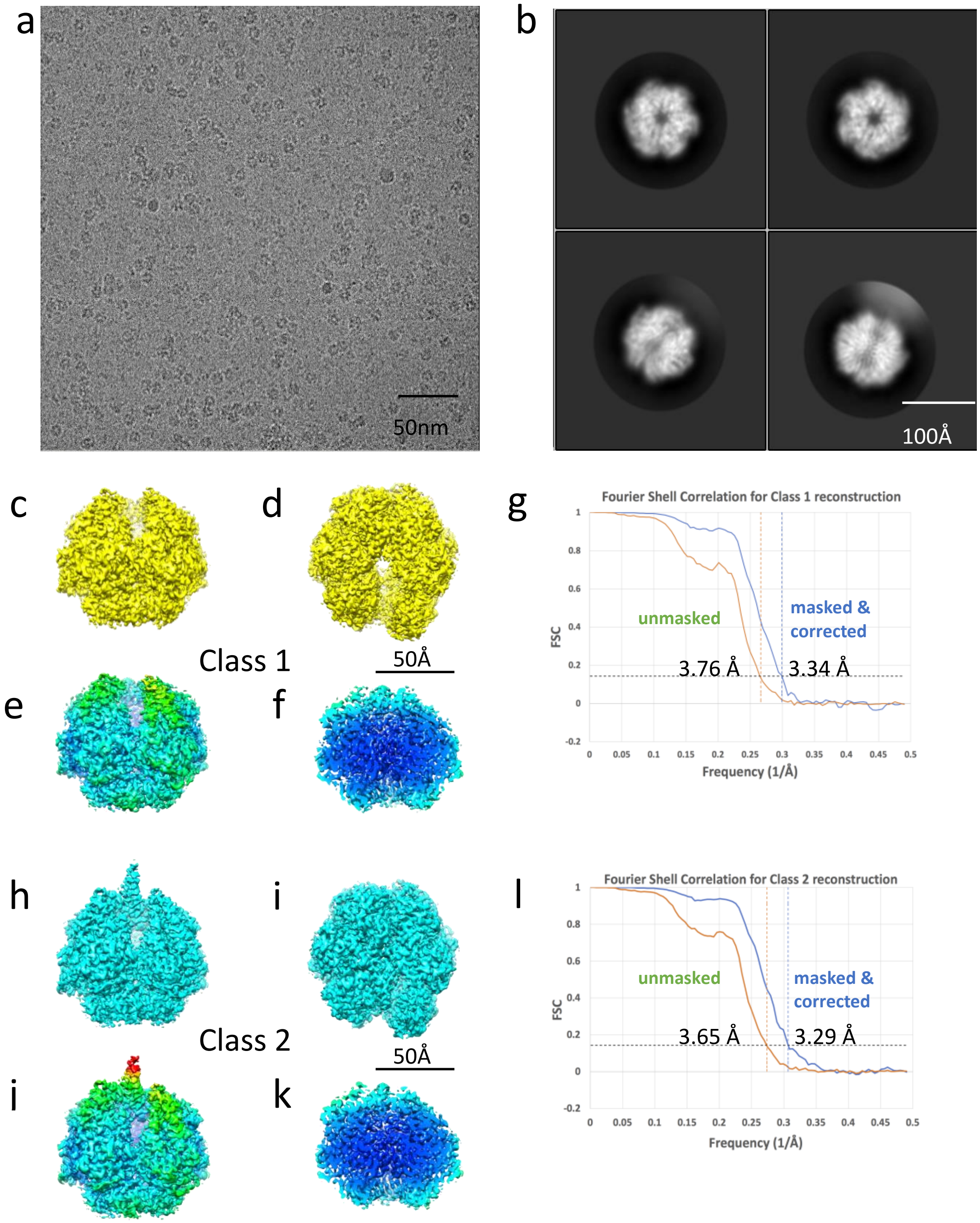

Supplementary Figure 3: CryoEM imaging and reconstruction of the EscN/EscO complex. (a) Representative micrograph. **(b)** Selected reference-free 2D class averages. **(c)** The top and **(d)** the side view of the class 1 reconstruction. **(e)** The side and **(f)** side-slab view of the class 1 reconstruction colored according to local resolution (blue – 3.2 Å, cyan – 3.8 Å, green – 4.4 Å, yellow – 5.0 Å, red – 5.6 Å). **(g)** FSC curve of the class 1 reconstruction using gold-standard refinement calculated from unmasked half maps and masked with the correction of soft masking effect. **(h)** The top and **(i)** the side view of the class 2 reconstruction. **(j)** The side and **(k)** side-slab view of the class 2 reconstruction colored according to local resolution as in **(e)** and **(f)**. **(l)** FSC curve of the class 2 reconstruction as in **(g)**.

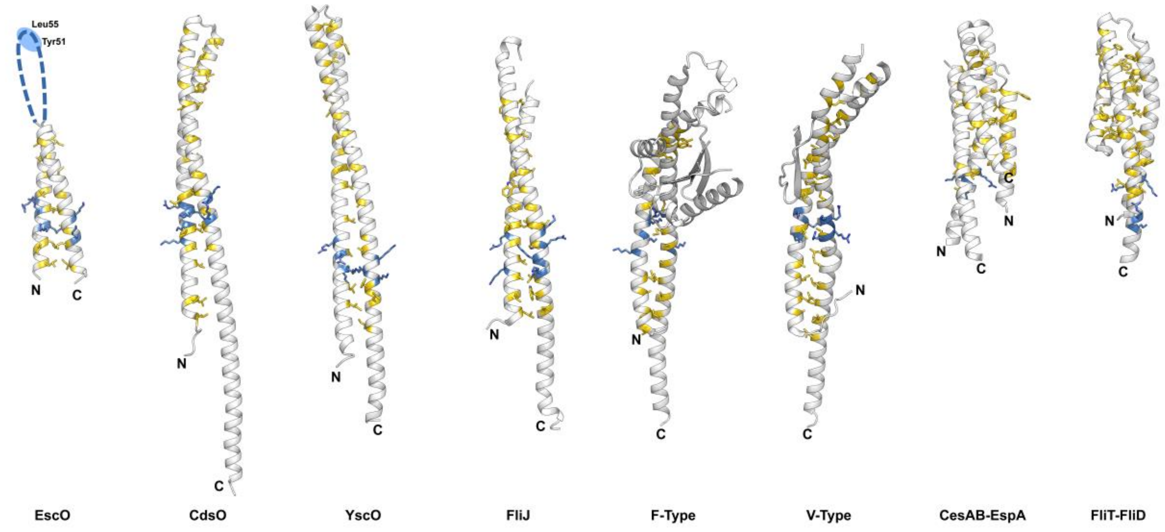

Supplementary Figure 4: EscO structural analysis. Comparison of EscO coiled coil secondary structure with characterized *v*T3SS homologues CdsO (PDB 3K29) and YscO (4MH6), *f*T3SS FliJ (3AJW), F_1 γ-subunit (1H8E), V_1 D subunit (3VR6), and chaperone-effector complexes CesAB/EspA (1XOU) and FliT-FliD (6CH2). A ring of positively charged residues, mirroring those found in EscO to be positioned at the EscN insertion interface, are shown in blue; hydrophobic residues facilitating the coiled coil are coloured yellow. The unresolved region of EscO is demarcated with a dotted line, and the approximate location of export gate interacting residues Tyr51 and Leu55 is shown in a blue circle.

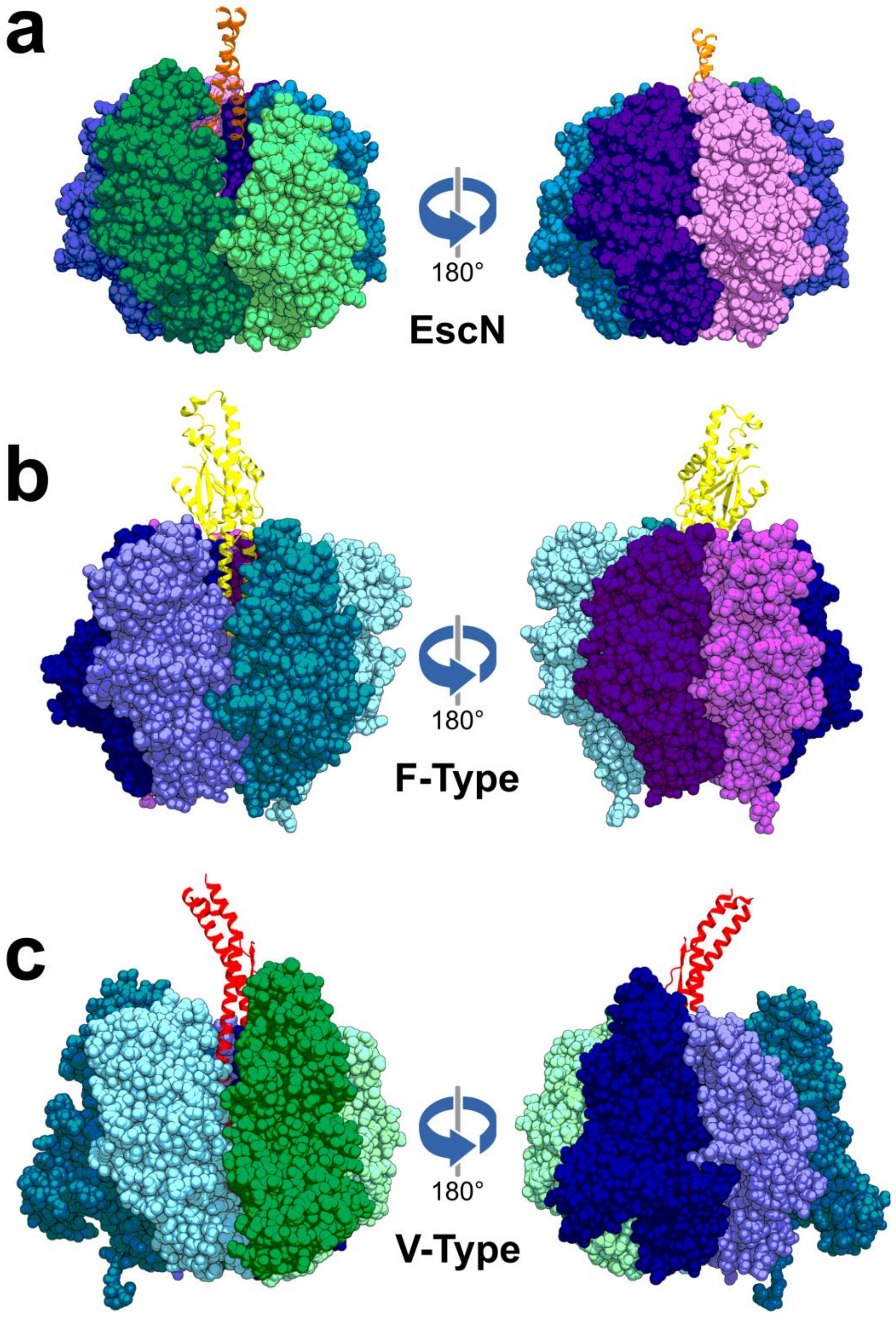

Supplementary Figure 5: Asymmetry of the EscN homohexamer. Comparison of quaternary structure of **(a)** EscN-EscO complex, **(b)** F-type ATPase $\beta_3\alpha_3\gamma$ complex (1H8E), and **(c)** V-type ATPase A_3B_3D complex (3VR6), showing the front view (left) with a prominent cleft in each structure versus comparatively tight packing in the rear view (right).

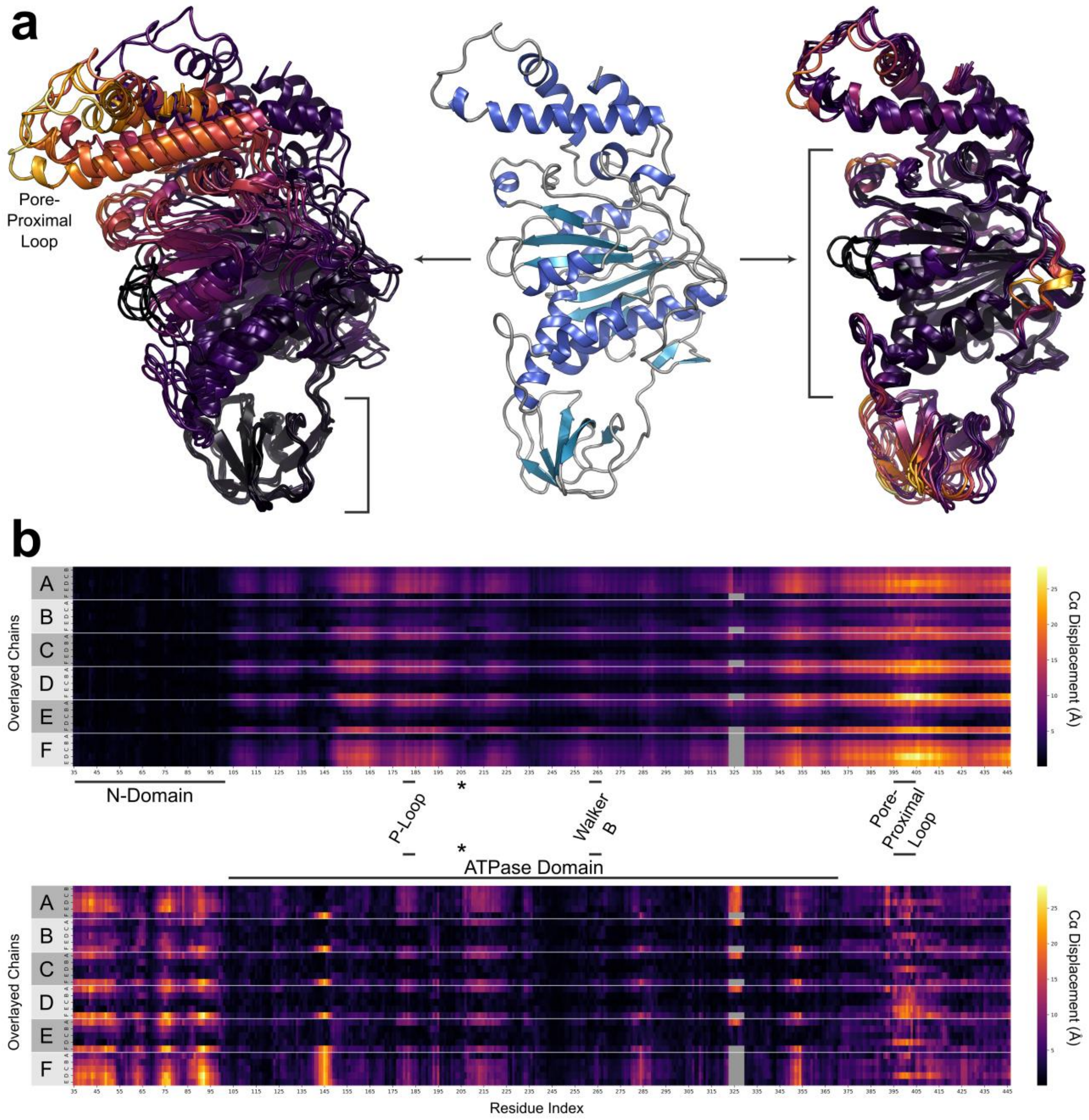

Supplementary Figure 6: Differences in conformation between EscN chains. (a) Alignment of EscN chains N_A through N_E on chain N_F, along the N-terminal domain (left) and the ATPase domain (right), coloured yellow in areas of high displacement between compared residues. **(b)** Heat maps of Cα displacement between residues when overlaying all combinations of EscN chains, aligned by the N-terminal domain (top) and ATPase domain (bottom). Locations of key motifs are labelled, with the catalytic glutamate highlighted with an asterisk.

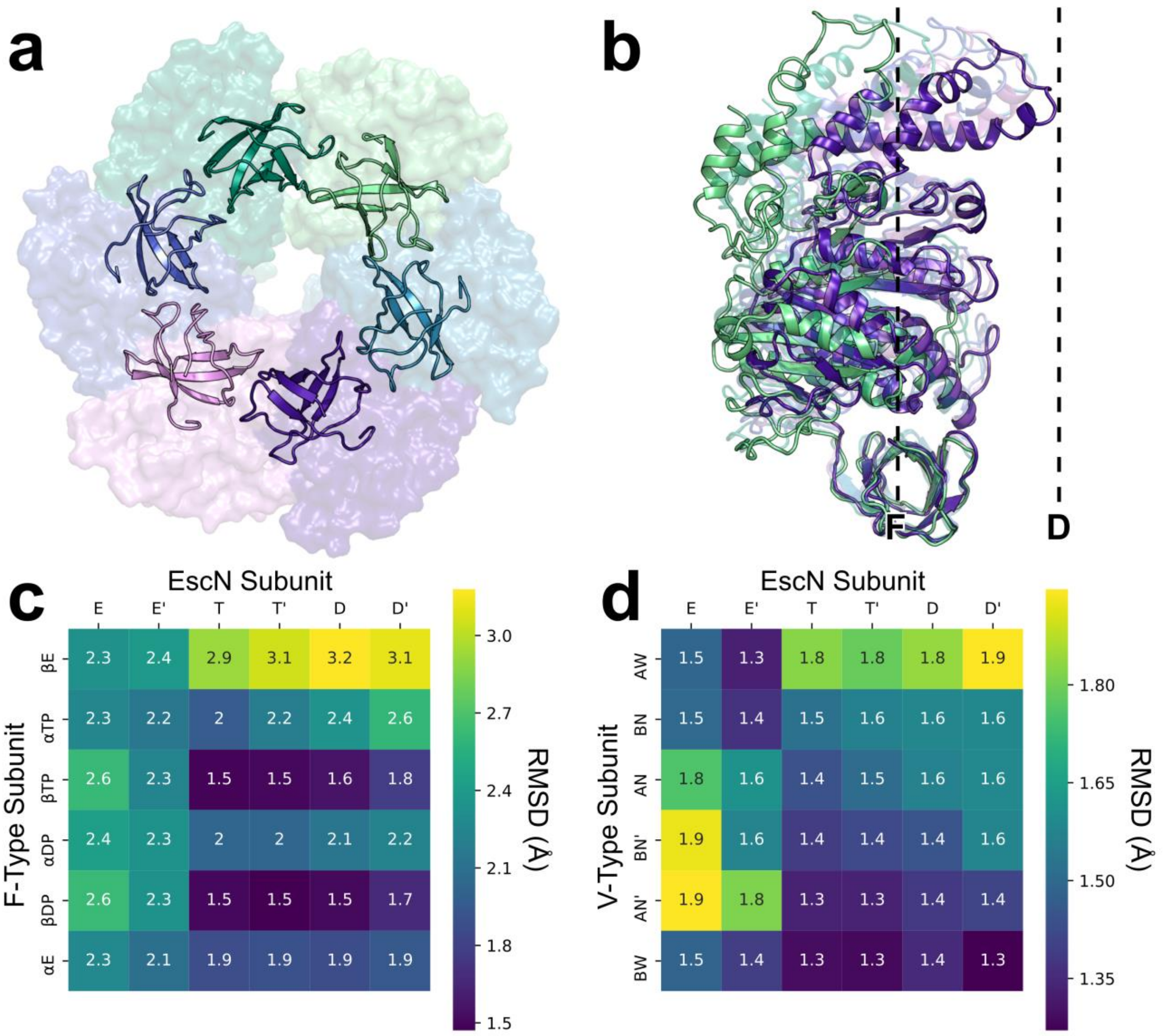

Supplementary Figure 7: EscN homohexamer formation and comparison with F- and V-ATPases. (a) Bottom view of the EscN class 1 N-terminal domains, highlighting their near-C6 symmetry. **(b)** Side view of EscN chains overlayed by their N-terminal domains, illustrating the strong tilt towards the pore by chain N_D (the D state) as compared to chain N_F (the E' state). **(c)** and **(d)** Heat maps of overall RMSDs comparing all ATPase catalytic conformations of EscN with those from F_1-ATPase (left, 1E1R) and V_1-ATPase (right, 3VR6).

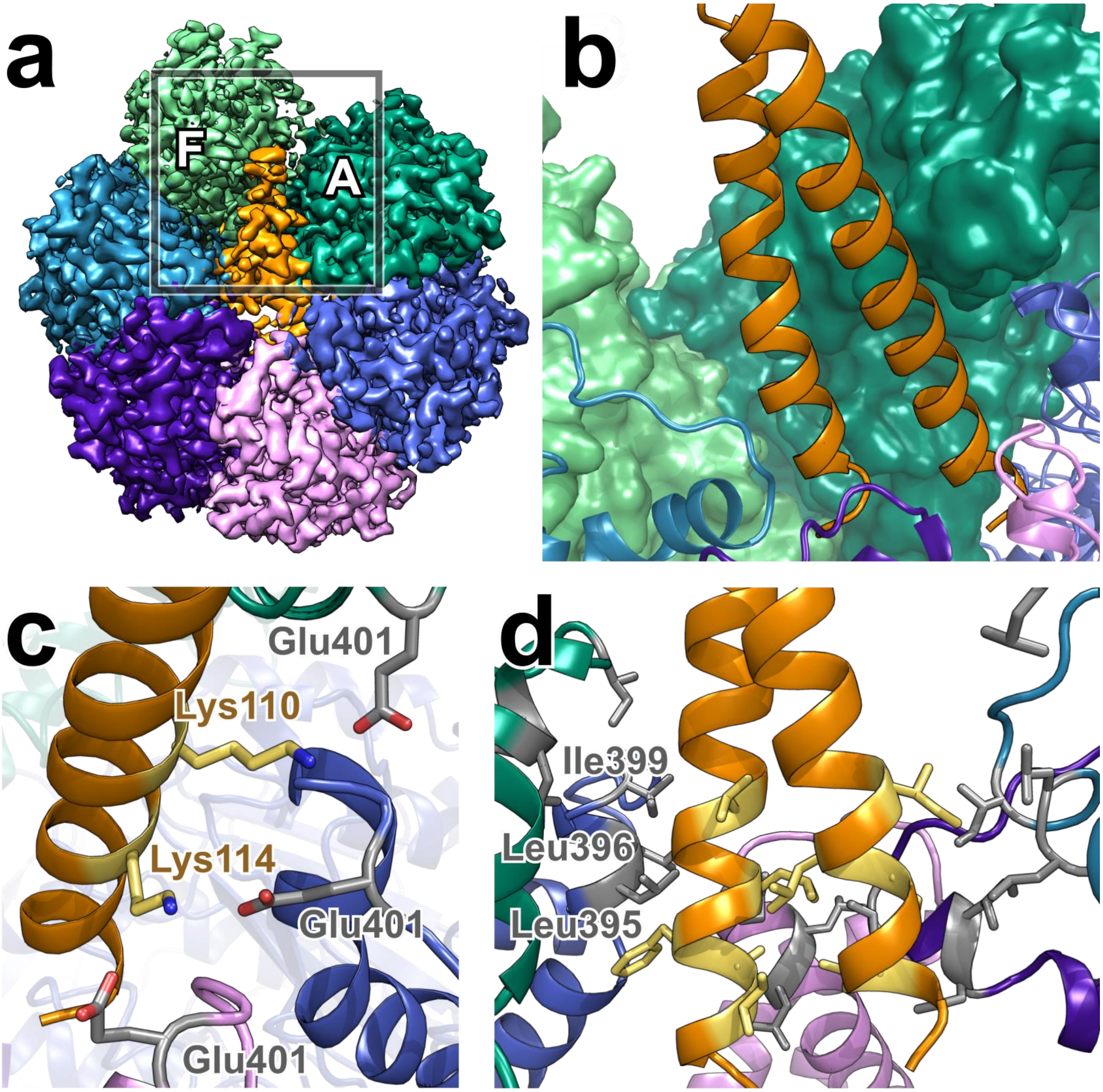

Supplementary Figure 8: EscO interaction interface with EscN. (a) Class 2 density of the EscN-EscO complex, demonstrating the relatively lower signal from chain N_F (the E' state), indicating higher dynamicity. **(b)** Binding pocket of EscO in between EscN chains N_F and N_A. **(c)** Interaction between EscN Glu401 from chains N_A, N_B, and N_C with EscO Lys110 and Lys114, an important interface for EscN-EscO complex formation and catalytic activity. **(d)** Hydrophobic ring of Leu395, Leu396, and Ile399 formed by EscN, which interacts with hydrophobic residues on EscO to stabilize it in the pore.

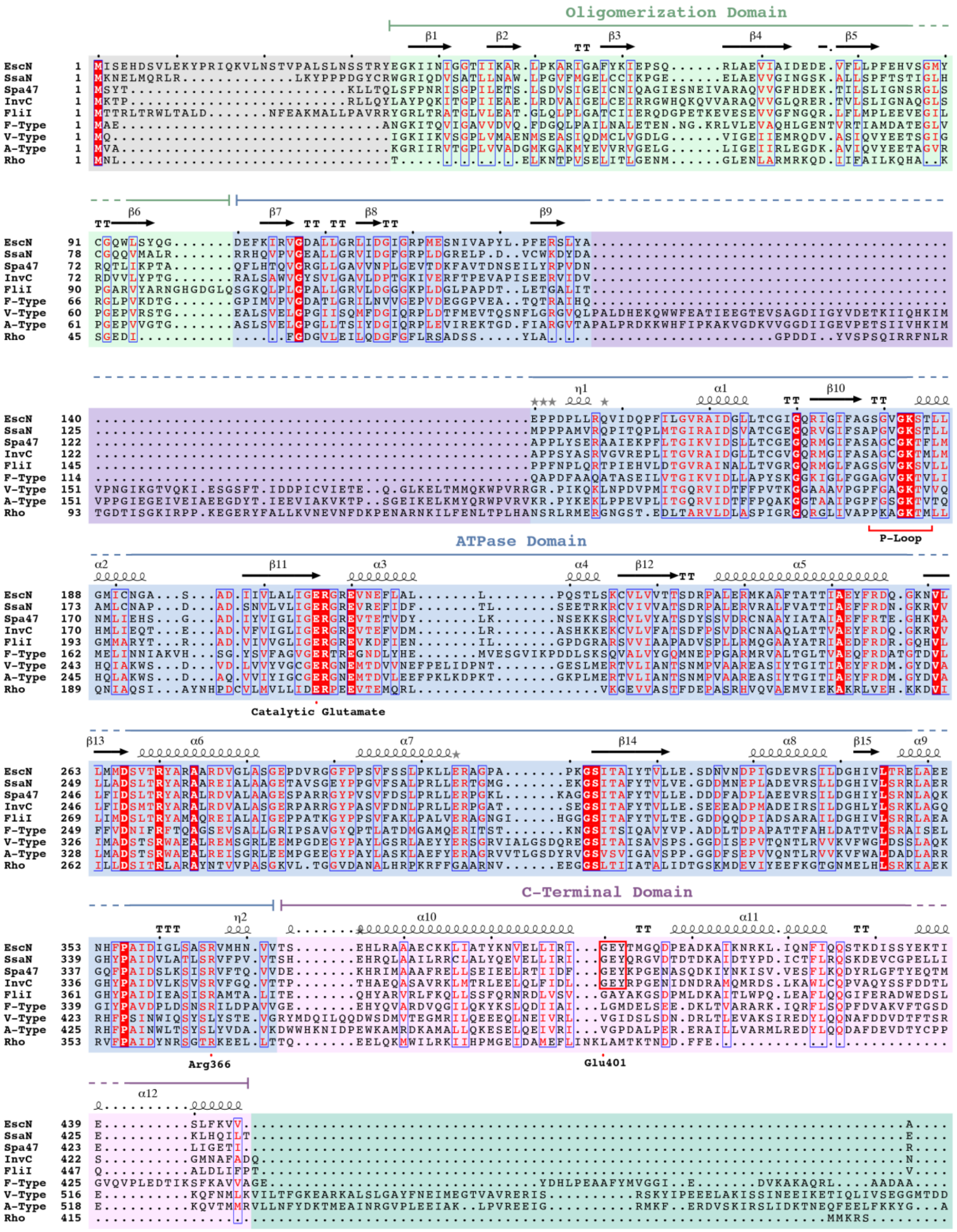

Supplementary Figure 9: Sequence alignment of EscN homologues. Sequence alignment by PSI-coffee of EscN with *v*T3SS homologues SsaN, Spa47, and InvC, *f*T3SS FliI, F_1 β catalytic subunit, V_1 A catalytic subunit, and Rho transcription termination factor.

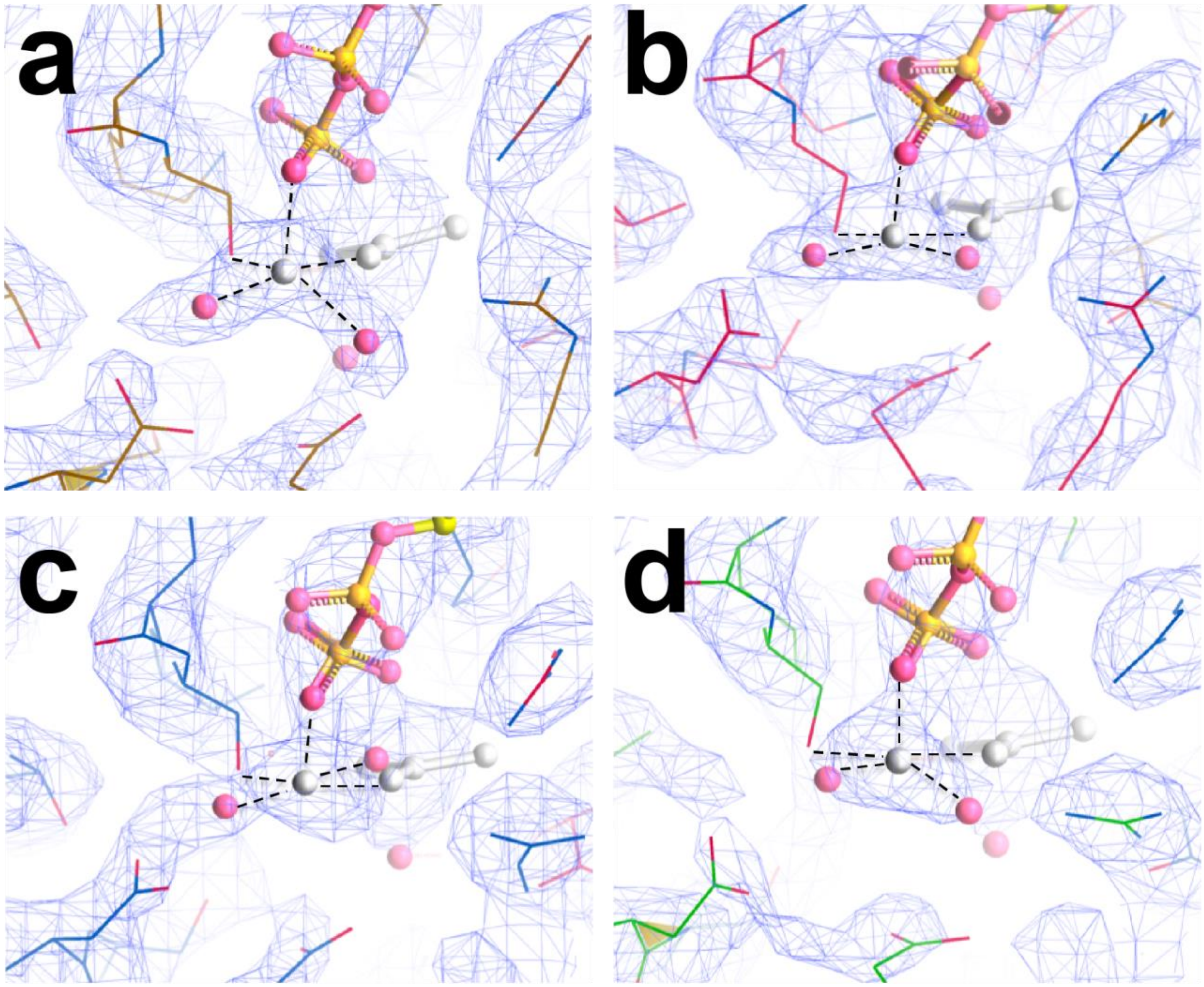

Supplementary Figure 10: Differences in magnesium ion coordination between the four ligand-bound active sites. The coordination moves from a trigonal bipyramidal geometry in **(a)** site T, with the waters migrating to reflect the expected octahedral geometry (with one water not resolved) in **(b)** site T', **(c)** site D, and **(d)** site D'.

Supplementary Table 1: List of primers

Primer Name	Sequence
EscN 29-446 Fwd	AAAAAACATATGAATTCGTCTACCAGATATGAAGGC
EscN 29-446 Rev	AAAAAACTCGAGCTCTCAATCGGTTCGCTCTGCTTT
EscO 1-125 Fwd	AAAAAACATATGTTGGACAGAATTTTATCTATTCGT
EscO 1-125 Rev	AAAAAAGGATCCATGGCCGAAAAGAAACAGGCTCTA
EscN E401A Fwd	GCTGCTTATTCGTATTGGTGCGTACACGATGGGGCAAG
EscN E401A Rev	CTTGCCCCATCGTGTACGCACCAATACGAATAAGCAGC
EscN L145R Fwd	GCTGAACCACCAGATCCTAGATTAAGGCAGGTTATTGATCAGCC
EscN L145R Rev	GGCTGATCAATAACCTGCCTTAATCTAGGATCTGGTGGTTCAGC
EscO K110E+K114E Fwd	CAAAAGAGAATCTGCAACATGTTAATGAATCAGTAGAAGAATTATCTTTTGCCATAAAGG
EscO K110E+K114E Rev	CCTTTATGGCAAAAGATAATTCTTCTACTGATTCATTAACATGTTGCAGATTCTCTTTTG
EscO R12E+R15E Fwd	CAGAATTTTATCTATTCGTAAAAGCGAAGCGAACGAATTGAGAGAATCAATGGCTAAG
EscO R12E+R15E Rev	CTTAGCCATTGATTCTCTCAATTCGTTCGCTTCGCTTTTACGAATAGATAAAATTCTG
