# Supplementary material for: Cryo-EM structure of the homohexameric T3SS ATPase-central stalk complex reveals rotary ATPase-like asymmetry
Source: Nat Commun. 2019 Feb 7;10:626. doi: 10.1038/s41467-019-08477-7 (PMC6367419; doi:10.1038/s41467-019-08477-7)
Supplement: Supplementary file 4 — Description of Additional Supplementary Files [file 41467_2019_8477_MOESM4_ESM.docx]

**Title:** Supplementary Movie 1
**Description:** Animation of EscN rotary catalysis. A linear morph between the EscN conformations demonstrates the differing subunit tilts with each catalytic state and the points of nucleotide binding and release.
